# Supplementary material for: Nutritional Regimes Enriched with Antioxidants as an Efficient Adjuvant for IBD Patients under Infliximab Administration, a Pilot Study
Source: Antioxidants (Basel). 2022 Jan 8;11(1):138. doi: 10.3390/antiox11010138 (PMC8773281; doi:10.3390/antiox11010138)
Supplement: Supplementary file 1 [file antioxidants-11-00138-s001.zip › antioxidants-1498256-supplementary.pdf]

SUPPLEMENTARY MATERIAL

Table S1: Bromatological composition of the diet

Nutrient	Daily Intake	Nutrient	Daily Intake	Nutrient	Daily Intake
Alcohol (mg)	0.00	Selenium (µg)	44.88	Tyrosine (mg)	3053.42
Protein (g)	95.85	C4:0-C10:0 (g)	2.38	Phenylalanine (mg)	3652.50
Fat (g)	49.60	C12:0 lauric (g)	0.83	Tryptophan (mg)	791.87
Carbohydrate (g)	332.16	C14:0 myristic (g)	2.76	Polyalcohol (mg)	0.00
Amid (g)	248.72	C16:0 palmitic (g)	10.11	Animal Protein (g)	48.14
Oligosaccharides (g)	64.79	C18:0 stearic (g)	3.45	Vegetal Protein (g)	47.71
Total Fiber (g)	30.60	C20:0 arachidic (g)	0.03	Asparagine (mg)	0.00
Cholesterol (mg)	155.30	C22:0 behenic (g)	0.02	Glutamine (mg)	0.00
Saturated Fat (g)	20.31	C14:1 Myristoleic Acid (g)	0.06	Pyrimidine (mg)	0.00
Polyunsaturated Fat (g)	5.39	C16:1 Palmitoleic Acid (g)	0.87	Total Mineral (mg)	0.00
Monounsaturated Fat (g)	23.91	C18:1 Oleic (g)	1.24	Chlorine (mg)	0.00
Calcium (mg)	1254.80	C20:1 Eicosaenoic (g)	0.00	Chromium (µg)	0.00
Sodium (mg)	1528.13	C22:1 Erucic (g)	0.00	Fluorine (mg)	33.89
Potassium (mg)	3267.24	C18:2 Linoleic (g)	1.05	Iodine (µg)	150.72
Phosphorus (mg)	1550.30	C18:3 Linolenic (g)	0.13	Manganese (mg)	2.97
Iron (mg)	12.45	C20:4 Arachidonic (g)	0.14	Molybdenum (µg)	3.74
Zinc (mg)	18.24	C20:5 EPA (g)	0.01	Nickel (mg)	0.00
Folic Acid (µg)	528.83	C22:6 DHA (g)	0.03	Beta-carotene (mg)	8845.28
Niacin (mg)	15.15	Phytic Acid (g)	0.27	Alpha-tocopherol (mg)	3.62
Riboflavin (mg)	1.20	Lysine (mg)	5594.45	Vitamin K (µg)	65.49
Thiamin (mg)	1.11	Histidine (mg)	2156.18	Vitamin B5 (mg)	3.64
Vitamin A (µg)	3093.40	Arginine (mg)	4108.58	Vitamin B8 – Biotin (µg)	1.10
Vitamin B6 (mg)	1.95	Aspartic Acid (mg)	7008.88	Vitamin B12 (µg)	3.36
Vitamin C (mg)	150.41	Threonine (mg)	3026.57	Caffeine (mg)	0.00
Vitamin D (µg)	0.85	Serine (mg)	3857.69	Inulin (mg)	0.00
Vitamin E (mg)	11.46	Glutamic Acid (mg)	12,457.08	Taurine (mg)	0.00
Oxalic Acid (mg)	25.20	Proline (mg)	4890.48	Choline (mg)	0.00
Cellulose (g)	1.50	Glycine (mg)	2765.93	L-Carnitine (mg)	0.00
Purine (mg)	225.90	Alanine (mg)	3375.25	Bicarbonate (mg)	0.00
Water (g)	865.34	Cysteine (mg)	742.24	H-Orac (µmol)	9892.00
Insoluble Fiber (g)	18.00	Valine (mg)	3789.72	L-Orac (µmol)	162.00
Soluble Fiber (g)	3.48	Methionine (mg)	1887.60	Total-Orac (µmol)	9939.00
Magnesium (mg)	221.23	Isoleucine (mg)	3382.51	Total Polyphenols (mg)	914.80
Copper (mg)	2.35	Leucine (mg)	6076.85	Potential Renal Acid Load (mEq)	13.65

Table S2: Description of the physical and hematological data at the follow-up times (N:47). Statistically significant differences (p-value ≤ 0.05) are underlined in bold.

	T0		T3		p-value
	mean ± SD	median (min to max)	mean ± SD	median (min to max)	
Height (m)	1.66 ± 0.1	1.66 (1.47 to 1.88)	1.66 ± 0.1	1.66 (1.47 to 1.88)	--
Weight (Kg)	65.09 ± 14.15	61.9 (45.7 to 93.5)	65.09 ± 14.15	61.9 (45.7 to 93.5)	--
BMI (Kg/m^2)	23.83 ± 4.19	23.48 (17.43 to 34.44)	23.83 ± 4.19	23.48 (17.43 to 34.44)	--
Red Blood Cells (10^6 cells/µL)	4.74 ± 0.55	4.66 (3.68 to 6.32)	4.83 ± 0.56	4.81 (3.83 to 6.32)	0.09
Hemoglobin (g/L)	12.52 ± 1.9	12.35 (8.8 to 15.7)	12.82 ± 1.7	12.8 (9.5 to 15.9)	0.08
Transferrin (mg/dL)	256.04 ± 48.51	256.5 (140 to 350)	270.89 ± 55.94	265 (146 to 383)	**0.03**
Ferritin (ng/mL)	58.06 ± 66.12	41.4 (3.6 to 330.1)	55.09 ± 71.11	30.1 (1.7 to 311.7)	**<0.01**
Iron (%)	49.92 ± 30.2	45 (14 to 144)	70.04 ± 39.57	63 (10 to 206)	**<0.01**
Albumin (g/dL)	3.65 ± 0.47	3.65 (2.5 to 4.8)	3.88 ± 0.44	4 (2.7 to 4.6)	**<0.01**
Ceruloplasmin (mg/dL)	32.8 ± 12.09	30.6 (13.8 to 83)	29.05 ± 10.01	27.2 (12.5 to 58.9)	**<0.01**
Alpha-1 Antitrypsin (mg/dL)	219.44 ± 54.06	212.5 (136 to 370)	197.53 ± 51.05	189 (109 to 329)	**<0.01**
White Blood Cells (10^3/µL)	9.02 ± 3.33	8.45 (2.72 to 17.57)	7.12 ± 2.12	7.03 (3.78 to 17.76)	**<0.01**
Basophil (%)	0.42 ± 0.22	0.4 (0.1 to 1)	0.6 ± 0.32	0.6 (0.1 to 1.8)	**<0.01**
Basophil (10^3 cells/µL)	0.04 ± 0.02	0.03 (0.01 to 0.11)	0.04 ± 0.02	0.04 (0.01 to 0.09)	0.26
Eosinophil (%)	2.12 ± 2.04	1.4 (0.1 to 9.1)	2.61 ± 1.76	2.2 (0.3 to 8)	**0.01**
Eosinophil (10^3/µL)	0.2 ± 0.25	0.1 (0.01 to 1.28)	0.18 ± 0.14	0.14 (0.02 to 0.6)	0.19
Neutrophil (%)	65.9 ± 9.61	66.9 (42.3 to 85.3)	55.27 ± 10.63	55.2 (36.2 to 85.3)	**<0.01**
Neutrophil (10^3 cells/µL)	6.16 ± 2.64	5.87 (1.8 to 12.01)	4.03 ± 1.7	3.79 (1.43 to 10.4)	**<0.01**
Monocytes (%)	7.92 ± 2.11	7.3 (4.3 to 12.1)	8.7 ± 2.44	8.8 (2.4 to 13.7)	**0.02**
Monocytes (10^3 cells/µL)	0.72 ± 0.33	0.61 (0.29 to 1.97)	0.61 ± 0.26	0.58 (0.25 to 1.9)	**0.03**
Lymphocytes (%)	23.65 ± 8.72	23.45 (8.1 to 43.8)	32.83 ± 8.81	33.9 (11.7 to 46.8)	**<0.01**
Lymphocytes (10^3 cells/µL)	2.01 ± 0.73	1.9 (0.99 to 4.59)	2.19 ± 0.74	2.03 (0.14 to 4.84)	**<0.01**
Platelets (10^3 cells/µL)	338.67 ± 132.81	308 (157 to 893)	292.17 ± 103.92	272 (152 to 779)	**<0.01**

Table S3: Description of physical and hematological data in T3 according to the type of treatment. (N: 47). Statistically significant differences (p-value ⩽ 0.05) are underlined in bold.

	T3				
	**RFT**		**RED**		**p-value**
	mean ± SD	median (min to max)	mean ± SD	median (min to max)	
Height (m)	1.64 ± 0.11	1.61 (1.47 to 1.88)	1.67 ± 0.1	1.68 (1.49 to 1.83)	0.45
Weight (Kg)	65.4 ± 13.66	63.3 (45.7 to 93)	65.19 ± 15.29	61.15 (49 to 93.5)	0.90
BMI (Kg/m^2)	24.42 ± 4.42	24.27 (17.64 to 34.44)	23.25 ± 3.86	23.45 (17.43 to 29.77)	0.45
Red Blood Cells (10^6 cells/μL)	4.76 ± 0.56	4.81 (3.83 to 6.18)	4.92 ± 0.56	4.88 (4.1 to 6.32)	0.41
Hemoglobin (g/L)	12.91 ± 1.77	13.6 (9.5 to 15.3)	12.7 ± 1.63	12.6 (10.3 to 15.9)	0.53
Transferrin (mg/dL)	274.44 ± 48.69	277 (180 to 358)	266.1 ± 65.49	257.5 (146 to 383)	0.77
Ferritin (ng/mL)	45 ± 43.58	29.3 (3 to 161.7)	68.71 ± 96.45	30.6 (1.7 to 311.7)	0.87
Iron (%)	73.56 ± 36.3	69 (14 to 145)	65.3 ± 44.13	60.5 (10 to 206)	0.37
Albumin (g/dL)	3.88 ± 0.41	4 (3 to 4.4)	3.88 ± 0.49	3.95 (2.7 to 4.6)	0.82
Ceruloplasmin (mg/dL)	32.28 ± 11.08	32.7 (14 to 58.9)	24.67 ± 6.29	23.45 (12.5 to 37.6)	<u>**0.01**</u>
Alpha-1 Antitrypsin (mg/dL)	203.04 ± 54.32	189 (139 to 299)	190.1 ± 46.59	189 (109 to 329)	0.65
White Blood Cells (10^3/μL)	6.97 ± 1.62	7.05 (3.78 to 10.21)	7.33 ± 2.68	6.98 (4.52 to 17.76)	0.85
Basophil (%)	0.58 ± 0.27	0.6 (0.1 to 1.1)	0.62 ± 0.38	0.6 (0.2 to 1.8)	0.84
Basophil (10^3 cells/μL)	0.04 ± 0.02	0.04 (0.01 to 0.08)	0.04 ± 0.02	0.04 (0.01 to 0.09)	0.78
Eosinophil (%)	2.48 ± 2.02	1.9 (0.3 to 8)	2.77 ± 1.37	2.45 (0.7 to 5.6)	0.20
Eosinophil (10^3/μL)	0.17 ± 0.15	0.12 (0.02 to 0.6)	0.2 ± 0.12	0.18 (0.06 to 0.56)	0.09
Neutrophil (%)	55.35 ± 12.41	53.4 (36.2 to 85.3)	55.16 ± 7.92	57.25 (40.6 to 66.2)	0.85
Neutrophil (10^3 cells/μL)	3.98 ± 1.69	3.67 (1.43 to 8.71)	4.11 ± 1.76	3.84 (1.91 to 10.4)	0.57
Monocytes (%)	8.71 ± 2.84	8.8 (2.4 to 13.7)	8.7 ± 1.84	8.85 (4.4 to 11.9)	0.93
Monocytes (10^3 cells/μL)	0.58 ± 0.18	0.58 (0.25 to 0.97)	0.65 ± 0.34	0.6 (0.28 to 1.9)	0.69
Lymphocytes (%)	32.88 ± 10.36	34.4 (11.7 to 46.8)	32.76 ± 6.4	31.65 (21.1 to 44.5)	0.67
Lymphocytes (10^3 cells/μL)	2.09 ± 0.76	2.01 (0.14 to 3.36)	2.34 ± 0.7	2.25 (1.54 to 4.84)	0.34
Platelets (10^3 cells/μL)	265.67 ± 58.53	260 (152 to 450)	327.95 ± 138.25	309.5 (187 to 779)	0.07

Table S4: Description of physical and hematological data at follow-up observation (T3) in CD patients, according to the type of treatment. (N: 22). Statistically significant differences (p-value ≤ 0.05) are underlined in bold.

	CD				
	RFT		RED		
	mean ± SD	median (min to max)	mean ± SD	median (min to max)	p-value
Height (m)	1.66 ± 0.08	1.61 (1.57 to 1.81)	1.65 ± 0.11	1.67 (1.49 to 1.8)	0.66
Weight (Kg)	63.09 ± 12.39	63.3 (45.7 to 84.5)	68.27 ± 18.39	60.5 (49.9 to 93.5)	0.64
BMI (Kg/m^2)	22.77 ± 3.69	21.9 (17.64 to 28)	24.71 ± 4.13	25.22 (18.34 to 29.77)	0.25
Red Blood Cells (10^6 cells/uL)	4.87 ± 0.67	4.88 (3.89 to 6.18)	4.88 ± 0.56	4.88 (4.1 to 6.15)	0.99
Hemoglobin (g/L)	13.05 ± 1.67	12.6 (10 to 15.3)	12.21 ± 1.43	12.5 (10.3 to 14.4)	0.31
Transferrin (mg/dL)	277 ± 47.8	277 (195 to 345)	289.56 ± 67.89	258 (206 to 383)	0.50
Ferritin (ng/mL)	35.6 ± 29.39	22.9 (6.8 to 89.6)	61.66 ± 98.09	25.7 (4.4 to 305.2)	0.94
Iron (%)	74.54 ± 30.11	69 (28 to 124)	74.22 ± 58.23	63 (20 to 206)	0.57
Albumin (g/dL)	3.94 ± 0.3	4 (3.5 to 4.3)	3.92 ± 0.38	3.7 (3.5 to 4.5)	0.89
Ceruloplasmin (mg/dL)	34.85 ± 12.7	36.3 (17.5 to 58.9)	23.94 ± 7.53	23.2 (12.5 to 37.6)	0.06
Alpha-1 Antitrypsin (mg/dL)	200.31 ± 50.52	189 (143 to 299)	164.89 ± 31.09	172 (109 to 196)	0.17
White Blood Cells (10^3/μL)	7.28 ± 1.66	7.11 (3.95 to 10.21)	7.25 ± 0.97	7.2 (6.04 to 9.24)	0.94
Basophil (%)	0.58 ± 0.27	0.7 (0.1 to 0.9)	0.57 ± 0.27	0.6 (0.2 to 1.1)	0.73
Basophil (10^3 cells/μL)	0.04 ± 0.02	0.05 (0.01 to 0.07)	0.04 ± 0.02	0.04 (0.01 to 0.08)	0.99
Eosinophil (%)	2.44 ± 2.19	1.7 (0.3 to 8)	2.24 ± 1.58	1.8 (0.7 to 5.6)	0.81
Eosinophil (10^3/μL)	0.17 ± 0.16	0.13 (0.02 to 0.6)	0.16 ± 0.12	0.13 (0.06 to 0.41)	0.86
Neutrophil (%)	56.69 ± 14.12	55.2 (36.2 to 85.3)	57.32 ± 7.18	58.1 (48 to 66.2)	0.81
Neutrophil (10^3 cells/μL)	4.3 ± 1.99	4 (1.43 to 8.71)	4.17 ± 0.83	3.88 (2.97 to 5.9)	0.69
Monocytes (%)	8.56 ± 3.09	9.7 (2.4 to 12.4)	7.74 ± 1.8	8.4 (4.4 to 9.7)	0.27
Monocytes (10^3 cells/μL)	0.6 ± 0.22	0.58 (0.25 to 0.97)	0.56 ± 0.17	0.6 (0.28 to 0.79)	0.73
Lymphocytes (%)	31.75 ± 11.53	33.9 (11.7 to 46.8)	32.12 ± 5	30.7 (24.6 to 39.4)	0.92
Lymphocytes (10^3 cells/μL)	1.94 ± 0.78	2.01 (0.14 to 3.36)	2.31 ± 0.37	2.4 (1.83 to 2.92)	0.20
Platelets (10^3 cells/μL)	247.69 ± 44.34	250 (157 to 342)	300.78 ± 52.1	299 (221 to 367)	**0.03**

Table S5: Description of physical and hematological data at follow-up observation (T3) in UC patients, according to the type of treatment. (N: 25). Statistically significant differences (p-value ≤ 0.05) are underlined in bold.

	UC				
	RFT		RED		
	mean ± SD	median (min to max)	mean ± SD	median (min to max)	p-value
Height (m)	1.63 ± 0.13	1.6 (1.47 to 1.88)	1.68 ± 0.09	1.69 (1.54 to 1.83)	0.22
Weight (Kg)	67.56 ± 14.87	67 (46.3 to 93)	62.66 ± 12.57	61.8 (49 to 85)	0.50
BMI (Kg/m^2)	25.96 ± 4.61	24.59 (18.59 to 34.44)	22.06 ± 3.35	22.58 (17.43 to 27.68)	**<u>0.04</u>**
Red Blood Cells (10^6 cells/μL)	4.65 ± 0.42	4.78 (3.83 to 5.12)	4.96 ± 0.58	4.89 (4.38 to 6.32)	0.38
Hemoglobin (g/L)	12.79 ± 1.92	13.7 (9.5 to 14.9)	13.09 ± 1.74	12.7 (10.3 to 15.9)	0.78
Transferrin (mg/dL)	272.07 ± 51.18	275 (180 to 358)	246.91 ± 59.67	254 (146 to 335)	0.32
Ferritin (ng/mL)	53.72 ± 53.21	38.1 (3 to 161.7)	74.48 ± 99.49	32.8 (1.7 to 311.7)	0.89
Iron (%)	72.64 ± 42.39	74.5 (14 to 145)	58 ± 29.27	58 (10 to 127)	0.47
Albumin (g/dL)	3.84 ± 0.5	3.9 (3 to 4.4)	3.85 ± 0.58	4 (2.7 to 4.6)	0.91
Ceruloplasmin (mg/dL)	29.9 ± 9.16	29.25 (14 to 47.8)	25.26 ± 5.39	25.3 (14.4 to 33.6)	0.18
Alpha-1 Antitrypsin (mg/dL)	205.57 ± 59.41	208 (139 to 287)	210.73 ± 48.07	209 (158 to 329)	0.84
White Blood Cells (103/μL)	6.68 ± 1.59	6.54 (3.78 to 9.05)	7.41 ± 3.58	6.77 (4.52 to 17.76)	0.80
Basophil (%)	0.59 ± 0.29	0.55 (0.1 to 1.1)	0.66 ± 0.46	0.6 (0.3 to 1.8)	0.91
Basophil (10^3 cells/μL)	0.04 ± 0.02	0.04 (0.01 to 0.08)	0.04 ± 0.02	0.04 (0.02 to 0.09)	0.73
Eosinophil (%)	2.53 ± 1.94	2.05 (0.5 to 6.9)	3.2 ± 1.07	3.2 (2 to 5.1)	0.10
Eosinophil (10^3/μL)	0.17 ± 0.15	0.11 (0.04 to 0.55)	0.23 ± 0.12	0.19 (0.14 to 0.56)	**<u>0.02</u>**
Neutrophil (%)	54.11 ± 10.98	49.15 (42.6 to 73.5)	53.38 ± 8.39	56.9 (40.6 to 64.6)	0.89
Neutrophil (10^3 cells/μL)	3.67 ± 1.36	3.32 (1.61 to 6.65)	4.06 ± 2.31	3.76 (1.91 to 10.4)	0.76
Monocytes (%)	8.84 ± 2.7	8.65 (3.8 to 13.7)	9.48 ± 1.54	9.2 (7.1 to 11.9)	0.31
Monocytes (10^3 cells/μL)	0.57 ± 0.15	0.56 (0.34 to 0.84)	0.72 ± 0.42	0.6 (0.4 to 1.9)	0.44
Lymphocytes (%)	33.94 ± 9.46	38.2 (17.3 to 44.7)	33.28 ± 7.55	31.7 (21.1 to 44.5)	0.78
Lymphocytes (10^3 cells/μL)	2.23 ± 0.75	2.19 (1.12 to 3.34)	2.36 ± 0.91	2.11 (1.54 to 4.84)	0.80
Platelets (10^3 cells/μL)	282.36 ± 66.43	286.5 (152 to 450)	350.18 ± 181.48	320 (187 to 779)	0.45

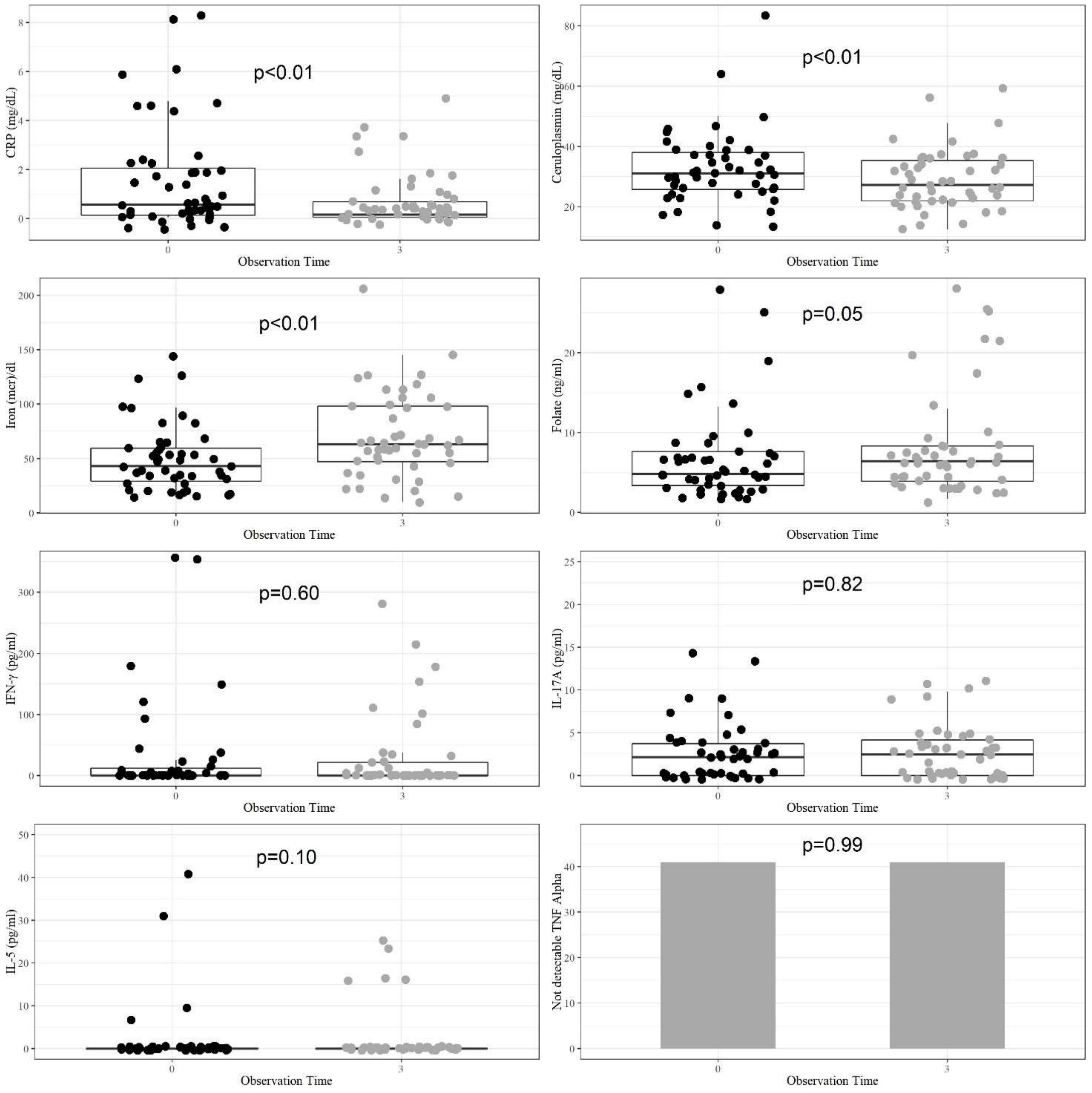

Figure S1: Serological biomarkers that were different between the two groups at T0 and T3 in all the enrolled subjects (N: 47)

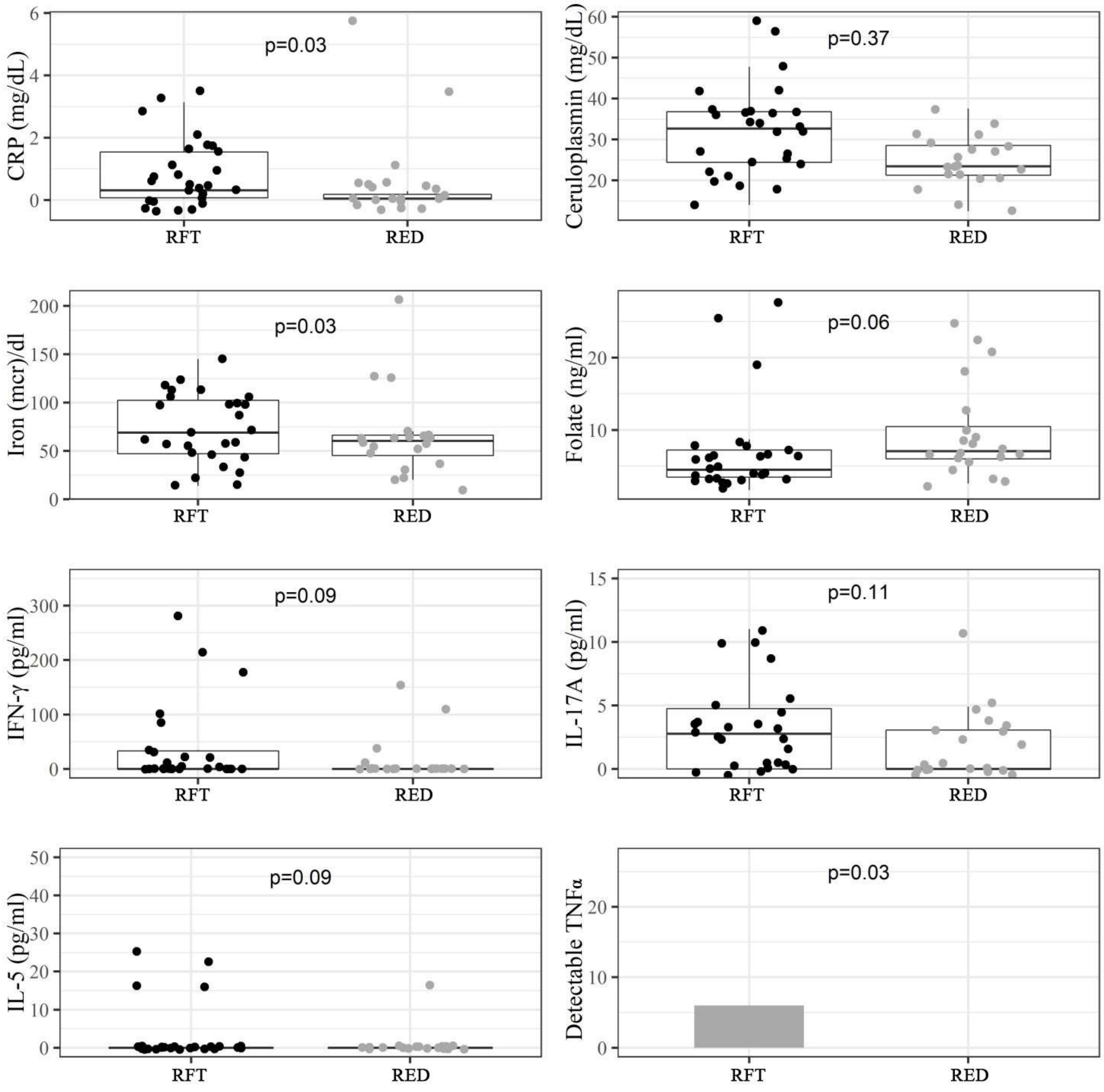

Figure S2: Serological biomarkers that were different between the red fruit tea (RFT) or purple corn extract (RED) groups at T3 (N: 47)

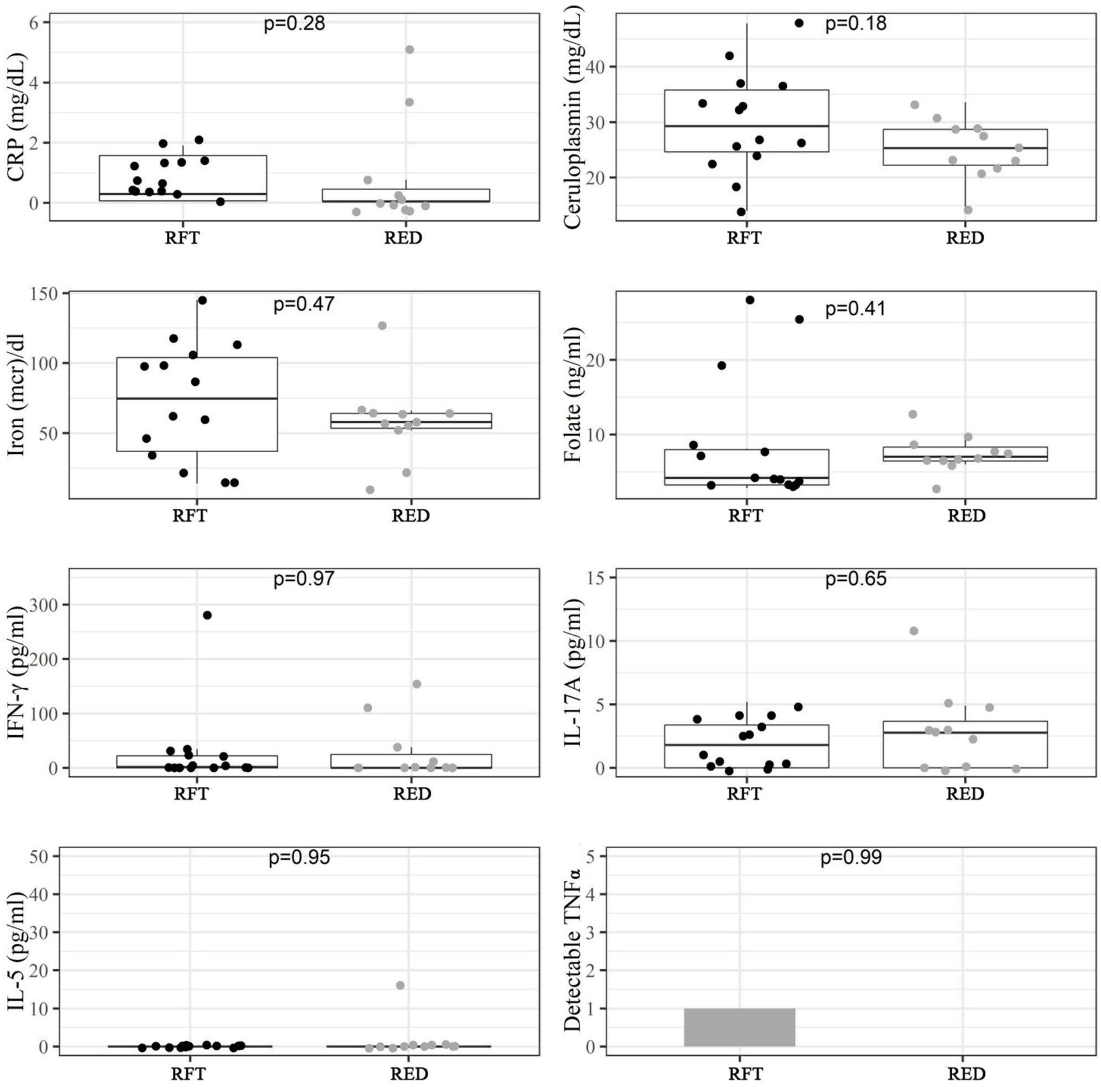

Figure S3: Serological biomarkers that were different between the red fruit tea (RFT) and purple corn extract (RED) groups in UC patients at T3 (N: 25)
